# Evaluating stress distribution and clinical success in varied dental implant designs

**DOI:** 10.6026/973206300220482

**Published:** 2026-01-31

**Authors:** Utkarsh Singh, Antriksh Azad, Prakhar Agarwal, Vidhi Chhabra Rathi, Anshul Jain, Kritika Sehrawat, Santosh Kumar

**Affiliations:** 1 Department of Dental Sciences, Apollo Medics Super Speciality Hospitals, Lucknow, India; 2 Department of Conservative Dentistry & Endodontics, College of Dental Science & Hospital, Rau, Indore, India; 3 Department of Oral Pathology and Microbiology, Darshan Dental College, Udaipur, India; 4 Department of Oral and maxillofacial surgery, Kalka Dental College, Meerut, India; 5 Department of Periodontology & Implantology, Karnavati University, Uvarsad, Gandhinagar, Ahmedabad, Gujarat, India

**Keywords:** Dental implants, stress distribution, finite element analysis, platform-switching, tapered implants, clinical success, marginal bone loss

## Abstract

Biomechanical implant design influences peri-implant stress distribution and long-term bone maintenance despite modern advances. Therefore, it is of interest to compare traditional threaded, tapered and platform-switched implants in 75 patients receiving 90 implants across varying bone densities and arch locations over 3 years. Platform-switched implants showed 34% lower crestal stress (p<0.001) versus conventional designs via FEA validation. Marginal bone loss measured 0.42±0.18 mm for platform-switched versus higher losses in others (p<0.001), with equivalent survival rates. Platform-switching provides superior biomechanical protection and bone preservation, establishing evidence-based design superiority for clinical practice.

## Background:

Dental implant therapy has changed into a reliable rehabilitation option to edentulous spaces and reported survival rates of over 95 percent in five year intervals [1]. Although such positive results were achieved, there are still biological and biomechanical issues, especially relating to marginal bone resorption and peri-implant tissue stability [2]. This interaction of the implant macro-geometry, surface topography and loading dynamics results in the formation of different stress patterns in the peri-implant bone which may impact on the long-term success [3]. More recent studies have shown that thread pitch, taper angle and platform-switching designs alter the distribution of strain at the bone-implant interface [4]. Nevertheless, the majority of the current literature use finite element analysis (FEA) with no clinical validation, which generates a gap in translatability between computational and clinical results [5]. Historically, standard threaded implants that have parallel walls are the best test, as they allow predictable osseointegration in sufficient volume of bone [6]. Tapered implant designs were developed to increase primary stability in weakened sites especially in extraction sockets and low-density bone [7]. Platform- switching dates back to the early 2000s, in which the size of an abutment is smaller than the implant neck, which allegedly generates less crestal bone stress due to biomechanical modulation [8]. Although both designs have theoretical strengths, there is a lack of head-to-head clinical studies that evaluate the two biomechanical parameters as well as clinical success indicators [9]. Literature available typically incorporates a number of design changes in individual cohorts, making the impact of macro geometry [10] separate. Moreover, the design-selection protocols on the basis of specific anatomical conditions of patients are not properly defined [11]. The importance of the design-specific performance is not limited to the realm of academic interest but it has an immediate effect on treatment planning, complication rates and economic healthcare burden [12]. Computation modeling indicates that foci of stress beyond physiological bone tolerance limits can initiate the activation of osteoclasts and eventual bone erosion [13]. Clinical observations have associated excessive micromotion during the healing process with fibrous encapsulation instead of the process of osseointegration [14]. The most recent systematic reviews also point to the methodological heterogeneity of the relevant implant studies, pointing to the necessity of a prospective trial, which would allow controlling confounding factors, including the surgical procedure, loading of the implant and the design of the prosthetic [15]. There is also a critical research gap in terms of the relationship between FEA-determined values of stress and the real marginal bone loss in controlled clinical settings [16]. Therefore, it is of interest to assess and compare three different implant designs namely conventional threaded, tapered and platform- switched through combining computational stress analysis with 36-month clinical outcomes.

## Materials and Methods:

## Study design and setting:

This is a prospective and parallel-group randomized and controlled clinical trial, which was carried out at the Department of Oral and Maxillofacial Surgery, January 2020-December 2023.

## Sample size calculation:

The determination of sample size was performed through a power analysis that was done on the basis of differences in marginal bone loss (primary outcome expected). Considering a standard deviation of 0.4 mm, clinically significant difference of 0.3 mm between groups, alpha of 0.05 and power of 0.80, 28 implants per group were needed. Taking into consideration the 10% attrition, the target population was determined to be 90 implants in three groups (n=30 in each group).

## Participant selection:

Seventy-five partially edentulous patients who needed a single-tooth replacement in either anterior or posterior maxilla or mandible were registered. The inclusion criteria included: age 25-70 years, bone height 10 mm and width 6 mm, the absence of uncontrolled systemic disease (ASA I-II) and smoking less than 10 cigarettes/day. The exclusion criteria were: Active periodontitis, radiation therapy on jaws, bruxism and night guard refusal, immunosuppressants and pregnancy. Intraoperative classification of bone density was based on Lekholm and Zarb (D1-D4).

## Randomization and implant systems:

Three titanium implants (4.1 mm in diameter, 10 mm in length), of commercial purity, were tested:

[1] Group A: Traditional threaded parallel-wall implant (ScrewVent, Zimmer Biomet)

[2] Group B: Tapered screw-vent implant ( Tapered ScrewVent, Zimmer Biomet )

[3] Group C: Parallel-wall implant that is platform-switched (Bone Level, Straumann) and parallel-wall.

Sealed envelopes were used to randomize blocks according to bone density (D1-D2 versus D3-D4) and location of arches (maxilla versus mandible). Each of the implants had the same surface topography (sandblasted, acid-etched) and implants were installed by one experienced surgeon.

## Surgical protocol:

Full thickness flaps under local anesthesia were raised by employing a minimal invasive approach. Osteotomies were made according to the manufacturer guidelines with drilling successively at 800 rpm under heavy irrigation. The surgical torque was measured at a torque controlled surgical motor during the implant insertion. Abutments (2 mm height) were inserted and flaps were repositioned without full coverage. The antibiotics prescribed were postoperative (amoxicillin 500 mg TID 5 days) and chlorhexidine rinse (0.12% BID 7 days). Any implants were done in accordance with a standard loading regimen after 12 weeks (mandible) or 16 weeks (maxilla).

## Finite element analysis:

The micro-CT scan (SkyScan 1272, Bruker) of the real implants were used to construct a three dimensional FEA model, which was made to each design. The models used literature values of cortical and trabecular bone properties (cortical: Youngs modulus 13.7 Gpa, Poisson ratio 0.30; Trabecular: 1.37 Gpa, 0.30). Functional loads were simulated by applying a 100 N axial load and a load of 50 N oblique load (30 o lingual). Mesh convergence test set optimum element sizes (0.2 mm). The values of stress (von Mises) were derived at crestal, apical and mid-implant levels.

## Clinical and radiographic examination:

The primary stability was evaluated by the values of insertion torque (Ncm) and resonance frequency (Osstell ISQ). Marginal bone loss was evaluated on standardized periapical radiographs by use of digital subtraction technique (ImageJ, NIH) at baseline, 6, 12, 24 and 36 months. The bleeding on probing and probing depth was noted at six sites on each implant. Every follow-up recorded some biological complication (peri-implant mucositis, peri-implantitis) and prosthetic complication (screw loosening, fracture). Success of implants was determined by modified Albrektsson criteria which included no mobility, radiographic bone loss of less than 1.5 mm/first year and less than 0.2 mm/subsequent years, no unremitting symptoms, no peri-implant infection.

## Statistical analysis:

The SPSS 26.0 was the version utilized in the analysis of data. Mean standard deviation was used to express continuous variables. Parametric data were compared using one-way ANOVA and post-hoc Tukey tests to compare inter-group differences. Categorical variables were determined using Chi-square tests. Cumulative survival rates were compared using Kaplan-Meier analysis of survival. Pearson correlation was used in the analysis of the correlations between marginal bone loss and FEA stress values. The level of statistical significance was determined as p<0.05.

## Results:

There were 90 implants on 75 patients (38 women, 37 men; Mean age of 52.3 years 8.7 years). The allocation of groups was done as 30 implants per group with equal distribution between bone density and arch location (Table 1). There were no major differences in the demographic parameters of groups (p). Four patients (5.3) lost to follow-up resulted in 86 final analysis implants being obtained. FEA revealed that patterns of stress distribution were different in designs (Table 2). Platform-switched implants also showed 34% reduced crestal stress concentration (28.4 ± 3.2 MPa) under the addition of an axial load as compared to conventional (43.1 ± 4.7 MPa, p<0.001) and 29% lower than tapered designs (39.8 ± 4.1 MPa, p<0.001). These differences were intensified by oblique loading and platform-switched implants reduced crestal stress by 41% (p<0.001). The level of stress at the apical areas were similar between groups (p=0.452). The difference between groups in terms of mean marginal bone loss at 36 months was significant (Table 3). Platform-switched implants showed 0.42 +/- 0.18 mm bone loss as compared to 0.89 +/- 0.31 mm in conventional (p=0.001) and 0.76 +/- 0.28 mm in tapered designs (p=0.002). The statistical significance of differences between conventional and tapered groups was not statistically significant (p=0.087). The Kaplan-Meier analysis of cumulative survival rates were 96.7% (29/30), 96.4% (27/28) and 100% (30/30) respectively of conventional, tapered and platform-switched implants. One traditional type of implant was unsuccessful at 8 weeks because no osseointegration had taken place and one tapered type of implant was taken out at 14 months because of peri-implantitis. There was no loss of platform switched implants. Log-rank test showed that there is no significant difference in the survival curves (p=0.784). The correlation analysis revealed that the mean stress of the crestal region of FEA and 36-month marginal bone loss showed a moderate positive relationship (r=0.68, p<0.001), indicating that the predictions of the computer programs are consistent with clinical phenomena.

## Discussion:

This randomized trial that offers both combined biomechanical and clinical data on the use of three modern dental implant designs. The main result indicates that, platform-switched implants considerably decrease the concentration of crestal stress and retain marginal bone with no deterioration to survival rate, whereas tapered designs provide better primary stability in defective bone. The biomechanical theories that support this reduction in crestal stress of 34% with platform-switching is that inward repositioning of the microgap and abutment connection dislodges stress off of the thin cortical crest [17]. This effect of stress shielding seems to have a clinical translation of a 53% decrease in marginal bone loss in comparison to traditional designs. The 0.42 mm average bone loss of the platform-switched group corresponds to the recent longitudinal studies that indicated a bone loss of less than 0.5 mm/3 years using this type of arrangement [18]. The moderate correlation of the FEA stress values and actual bone loss (r=0.68) confirms the use of computational modelling to make predictions, however, the correlation is not definite, indicating that additional biological processes play a role in bone remodelling. Tapered implants showed a higher mechanical engagement with 19% more insertion torque and ISQ values which would be beneficial in D3-D4 bone quality. This increased stability in the primary stage which leads to hypothetical earlier functional loading and lesser micromotion throughout the healing process [19]. This mechanical advantage, however, failed to translate into bone preservation over conventional designs, whereby both groups had similar crestal bone loss patterns. The similarity between the distribution of stress of both taper and conventional designs under axial loading implies that the taper angle does not have a significant impact on the final load transfer behavior, but only at first. A high success rate of more than 96% in all groups confirms that contemporary implants designs are highly predictable provided that there is standardization in the surgical protocols and on the selection criteria of the patients.

The statistically insignificant difference between the survival rates sends the message that macrogeometry can have an effect on the biological parameters, without an impact on the overall survival, especially in situations where the prosthetic loading does not exceed physiological thresholds [20]. The only failure in the conventional group was early and could probably be explained by the surgical trauma instead of a design flaw and the fact that the tapered implant was lost to peri-implantitis at 14 months demonstrates that design alone cannot be more important than biological risk factors. The lowest levels of biological complications were observed in the platform-switched (3.2%), but the difference was not statistically significant because of the small sample size. This pattern demonstrates the assumption that inflammatory infiltrate caused by microgap is less and leads to better health of the peri-implant area [21]. The probe depths were much shallower in platform-switched implants, which may be due to less tissue inflammation and not loss of attachment as the marginal bone levels are exactly better. The merit of this study is that it uses a combined method with and without the use of computational modelling and prospective clinical data, randomizing the confounders and standardized protocols. This trial has compared forecasts of stress with radiography results unlike earlier works which extrapolated FEA results to clinical conditions without validation [22]. Moreover, stratification according to bone density provided equal distribution of anatomical challenges among groups. There are a few limitations that should be considered. Although the 36 months follow up is adequate to evaluate early remodelling of the bones, it fails to detect long term prosthetic complications and loss of bone after three years. The research was restricted to single-tooth replacements; the findings might not be applicable to splinted and full-arch reconstructions where there is significant variation in the distribution of loads [23]. Also, the topography of surfaces, though comparable across groups, was not the same, which is also a possible confounding factor. The single-operator protocol adds to uniformity, although it might not be valuable in generalizing to other levels of surgical skill. The study ought to be carried out further to 5-10 years of follow-up to determine lasting bone stability and complications of the prosthetics. The use of multi-center trials would strengthen generalizability and micro-CT analysis of the retrieved specimens would be effective to validate histomorphometrically FEA models. Design selection algorithms can be further optimized through investigation of patient-specific variables including the magnitude of bite forces and the use of parafunctional habits. These findings clinically go in favor of design-specific treatment planning. The platform-switched implants seem to be ideal in the aesthetic areas where bone conservation is of utmost importance whereas tapered designs can be used in extraction sockets or in low-density bone. In general locations where the quality of bones is sufficient, conventional parallel-wall implants can still be used. The similar existence rates indicate that the analysis of cost-effectiveness needs to consider the bone maintenance and complication rates as opposed to the price of an implant.

## Conclusion:

Platform-switched implants significantly reduce crestal stress by 34% and marginal bone loss by 53% versus conventional threaded designs. Tapered implants enhance primary stability in low-density bone without compromising long-term outcomes.

## Figures and Tables

**Table 1:** Baseline demographics and surgical parameters

**Parameter**	**Conventional (n=30)**	**Tapered (n=30)**	**Platform-Switched (n=30)**	**p-value**
Age (years)	53.1± 9.2	51.8± 8.4	52.0± 8.9	0.823
Sex (F/M)	15/15	16/14	17/13	0.851
Maxilla/Mandible	16/14	15/15	17/13	0.823
D1-D2/D3-D4 bone	18/12	17/13	19/11	0.851
Insertion torque (Ncm)	32.4± 6.8	38.7± 7.2	33.1± 6.5	0.003*
ISQ value	72.3± 5.1	76.8± 4.9	73.0± 5.3	0.004*
*Statistically significant difference
(p<0.05). Post-hoc analysis revealed
tapered group demonstrated significantly
higher insertion torque and ISQ values
compared to conventional and
platform-switched groups

**Table 2:** Finite element analysis stress distribution (MPa)

**Loading Condition**	**Location**	**Conventional**	**Tapered**	**Platform-Switched**	**p-value**
Axial (100 N)	Crestal	43.1± 4.7	39.8± 4.1	28.4± 3.2	<0.001*
Axial (100 N)	Mid-implant	18.2± 2.3	19.5± 2.6	17.8± 2.1	0.089
Axial (100 N)	Apical	22.7± 3.1	23.4± 3.3	21.9± 2.9	0.452
Oblique (50 N)	Crestal	67.3± 6.8	62.1± 6.2	39.7± 4.5	<0.001*
Oblique (50 N)	Mid-implant	31.4± 3.8	33.2± 4.1	29.8± 3.5	0.067
Oblique (50 N)	Apical	28.6± 3.4	29.8± 3.7	27.5± 3.2	0.278
*Statistically significant
difference (p<0.05).
Post-hoc Tukey test confirmed
platform-switched group differed
significantly from both other groups

**Table 3:** Clinical outcomes at 36-month follow-up

**outcome measure**	**conventional (n=29)**	**tapered (n=28)**	**platform-switched (n=29)**	**p-value**
Marginal bone loss (mm)	0.89± 0.31	0.76± 0.28	0.42± 0.18	<0.001*
Probing depth (mm)	3.2± 0.6	3.1± 0.5	2.8± 0.4	0.023*
Bleeding on probing (%)	13.8	10.7	6.9	0.412
Biological complications (%)	10.3	7.1	3.2	0.389
Prosthetic complications (%)	6.9	3.6	3.2	0.589
Cumulative survival rate (%)	96.7	96.4	100	0.784
*Statistically significant difference
(p<0.05). Platform-switched group
demonstrated superior bone preservation
and reduced probing depths
